# Immunomodulatory properties of mesenchymal stem cells within three-dimensional collagen matrices

**DOI:** 10.1007/s11626-025-01109-z

**Published:** 2025-09-15

**Authors:** Yenny Yustisia, Koichi Kato

**Affiliations:** 1 Department of Biomaterials, Graduate School of Biomedical and Health Sciences, Hiroshima University, 1-2-3, Kasumi, Minami-ku, Hiroshima, 734-8553 Japan; 2 Department of Oral Biology, Faculty of Dentistry, University of Jember, Kalimantan 37, Jember, Indonesia

**Keywords:** Mesenchymal stem cells, Collagen hydrogel, Immunomodulation, Three-dimensional cell culture, Mechanotransduction, Inflammatory microenvironment

## Abstract

Mesenchymal stem cells (MSCs) hold promise for treating inflammatory and immune-related diseases; however, their clinical application is limited by poor survival and function post-transplantation. Collagen hydrogels may support MSC viability and function by mimicking the extracellular matrix. This study aimed to evaluate how cell density and collagen concentration within three-dimensional (3D) collagen matrices affect the immunomodulatory behavior of MSCs under inflammatory conditions. MSCs were embedded in collagen hydrogels of varying stiffness and seeded at different densities. Constructs were stimulated with proinflammatory cytokines (tumor necrosis factor-α and interferon-γ), and changes in Gene expression, hydrogel contraction, and cell viability were analyzed. Lower collagen concentrations and higher seeding densities enhanced MSC immunomodulatory Gene expression and matrix contraction. High cell density increased contraction but reduced cell viability in softer gels. Mechanical properties of the matrix, such as stiffness and viscoelasticity, influenced cell behavior via mechanotransduction pathways. Both physical and biological cues within 3D collagen hydrogels significantly regulated MSC immunomodulatory responses. Optimizing collagen concentration and seeding density may improve the therapeutic potential of MSC-based treatments.

**Supplementary Information:**

The online version contains supplementary material available at 10.1007/s11626-025-01109-z.

## Introduction

Mesenchymal stem cells (MSCs) have drawn considerable interest in regenerative medicine due to their therapeutic potential across a wide range of diseases, including musculoskeletal injuries, cardiovascular disorders, autoimmune conditions, and neurodegenerative diseases (Farini *et al*. 2014; Pittenger *et al*. 2019; Rodríguez-Fuentes *et al*. 2021). While initially explored for their capacity to differentiate and replace damaged cells, MSCs are now more broadly recognized for their ability to modulate the tissue microenvironment. Their anti-inflammatory, immunoregulatory, and pro-repair functions have been extensively characterized (Prockop and Oh, 2012); Bernardo and Fibbe 2013; Wu *et al*. 2020), making them attractive candidates for treating inflammation-driven pathologies.

The therapeutic efficacy of MSCs is largely attributed to paracrine signaling, through which they secrete a complex array of trophic and immunomodulatory factors. In response to inflammatory cues, MSCs release molecules such as prostaglandin E2 (PGE2), tumor necrosis factor–stimulated Gene 6 (TSG6), indoleamine 2,3-dioxygenase (IDO), and vascular endothelial growth factor (VEGF), which collectively orchestrate immune suppression and tissue regeneration (Jiang and Xu 2020; Sagaradze *et al*. 2020). For instance, PGE2 promotes macrophage polarization toward an anti-inflammatory M2 phenotype, inhibits natural killer (NK) cell activity, and enhances regulatory T cell differentiation (Vasandan *et al*. 2016; Galland *et al*. 2017; Burand *et al*. 2020). IDO suppresses T cell proliferation, while TSG6 reduces monocyte and macrophage recruitment (Harrell *et al*. 2021; Lu *et al*. 2021). VEGF, in turn, promotes angiogenesis and endothelial cell function (Ge *et al*. 2018; Kozhukharova *et al*. 2022).

Despite encouraging preclinical results, clinical application of MSCs remains challenging. A key limitation is the poor retention and survival of transplanted cells. Many studies report that most MSCs are lost within hours after administration, with less than 5% remaining at the target site (Levit *et al*. 2013). Transplanted cells face harsh conditions such as hypoxia, nutrient scarcity, and the absence of a supporting extracellular matrix (ECM). Moreover, as adherent cells, MSCs are vulnerable to anoikis—cell death triggered by ECM detachment—when delivered into suspension or non-adherent environments (Marquardt and Heilshorn 2016). Immunological rejection of allogeneic MSCs also remains a concern, as donor major histocompatibility complex (MHC) molecules can activate host T and NK cells or induce antibody responses (Zangi *et al*. 2009; Ankrum *et al*. 2014).

To address these limitations, hydrogel-based biomaterials have been explored as protective and supportive delivery vehicles for MSCs. Among them, collagen hydrogels are especially promising due to their biocompatibility, ECM-like structure, biodegradability, and mechanical tunability (Sarrigiannidis *et al*. 2021; Troy *et al*. 2021). Injectable formulations enable minimally invasive application, while the soft, porous network promotes nutrient diffusion and cellular viability (Burdick *et al*. 2016; Huang *et al*. 2022). Recent studies have shown that collagen hydrogels improve MSC retention and therapeutic performance without eliciting significant adverse responses (He *et al*. 2020; Wong *et al*. 2023).

However, the influence of collagen hydrogels on MSC function under inflammatory conditions remains poorly understood. In three-dimensional (3D) environments, physical interactions between cells and the surrounding matrix significantly affect cellular behaviors such as proliferation, gene expression, and immunomodulation (Chung *et al*. 2021; Zhang *et al. *2021). Thus, understanding how specific parameters—such as collagen concentration and cell density—modulate MSC responses is essential for optimizing hydrogel-based therapies. In this study, we investigated the effects of initial collagen concentration and MSC density on their immunomodulatory function and viability in inflammatory environments. Our findings provide insights into the design of more effective MSC delivery strategies using collagen-based scaffolds.

## Materials and methods

### Cell culture

Human bone marrow–derived MSCs were purchased from Lonza (PT-2501, Lot No. 22TL301024) and cultured in Dulbecco’s modified Eagle medium (DMEM)–low glucose with l-glutamine (Fujifilm Wako Pure Chemical, Osaka, Japan), supplemented with 10% heat-inactivated fetal bovine serum (FBS; Sigma Life Science, Burlington, MA), 1% penicillin–streptomycin solution (Nacalai Tesque, Kyoto, Japan), and 1 ng/mL recombinant human basic fibroblast growth factor (bFGF; Gibco Life Technologies, Carlsbad, CA). Cells were maintained at 37°C in a humidified atmosphere under 5% CO₂, and the culture medium was replaced every 3 d. When cultures reached approximately 70% confluency, MSCs were passaged using 0.25% trypsin–EDTA (Nacalai Tesque) and seeded at a density of 6,000 cells/cm^2^. MSCs at passage 6 were used for all subsequent experiments.

### Collagen hydrogel preparation and cell incorporation

Collagen hydrogels were prepared using bovine dermis-derived atelocollagen (AteloCell IPC-50; Koken, Tokyo, Japan), which was neutralized by mixing with 10 × DMEM (Sigma-Aldrich, St. Louis, MO), 7.5% NaHCO_3_ solution (Sigma-Aldrich), 1 M NaOH, and ultrapure water to yield a physiologically neutral collagen solution. To investigate the effects of culture dimensionality, neutralized collagen solutions at 3.5 mg/mL were mixed with human MSCs (hMSCs) at a density of 3 × 10⁶ cells/mL. hMSCs were also cultured on polystyrene plates coated with the same concentration of collagen hydrogel, as well as on uncoated plates, at a density of 20,000 cells/cm^2^ for comparison. To examine the effects of cell density, neutralized collagen solutions (3.5 mg/mL) were mixed with hMSCs to achieve final cell densities of 1 × 10^6^, 3 × 10^6^, 5 × 10^6^, and 7 × 10^6^ cells/mL. To assess the effects of collagen concentration, collagen solutions at 3.0, 3.5, and 4.0 mg/mL were each mixed with hMSCs at a constant density of 1 × 10^6^ cells/mL (low seeding density) and 5 × 10^6^ cells/mL (high seeding density). The resulting collagen–MSC mixtures were dispensed into 96-well tissue culture plates (100 µL per well) and incubated at 37°C in a humidified incubator for 1 h to allow gelation. Following gelation, the MSC–collagen constructs were incubated in low-glucose DMEM supplemented with 1% FBS, 2 mM l-glutamine, and 1% penicillin–streptomycin solution. To induce an inflammatory environment, the culture medium was further supplemented with 10 ng/mL tumor necrosis factor-α (TNF-α) and 25 ng/mL interferon-γ (IFN-γ) both from PeproTech, Cranbury, NJ. The inflammatory medium was replaced every 2 d. Constructs were harvested after 24 h and 5 d of culture for further analysis.

### Mechanical characterization

Collagen hydrogels at different concentrations (3.0, 3.5, and 4.0 mg/mL) without cells were prepared in 24-well polystyrene plates (well diameter, 15.5 mm; thickness, 5 mm) and incubated for 2 h to allow gelation. Dynamic mechanical analysis was performed at room temperature using a rheometer (RE 33005B; Yamaden, Tokyo, Japan). A continuous compressive force of 0.01 N was applied to each hydrogel at a rate of 1 mm/s. The elastic modulus was determined from the initial linear portion of the stress–strain curve.

### Quantification of hydrogel contraction

MSCs–collagen hydrogels in 96-well polystyrene plates were imaged using a digital scanner (SHARP BP-60C26, Sharp Corp., Osaka, Japan) on days 1, 3, 5, and 7. The surface area of the top view of each hydrogel was measured from the images using ImageJ software (National Institutes of Health, Bethesda, MD). Hydrogel contraction was calculated as the percentage reduction relative to the initial surface area.

### Cell viability

MSCs-incorporated collagen hydrogels were prepared in 96-well polystyrene plates. Cell viability was assessed at 24 h and 5 d post-incorporation using the Cell Counting Kit-8 (CCK-8; Dojindo, Kumamoto, Japan). Briefly, the culture medium was replaced with 200 µL of medium containing 10% CCK-8 solution, followed by incubation for 2 h. The absorbance of the supernatant was then measured at 450 nm using a microplate reader (Multiskan FC, Thermo Fisher Scientific, Tokyo, Japan). For calcein–propidium iodide (PI) staining, hydrogels were washed three times with phosphate-buffered saline (PBS), 15 min per wash, and then incubated for 20 min with PBS containing 2 µg/mL calcein-AM and 3 µg/mL PI (both from Dojindo). After staining, the hydrogels were washed with PBS. Fluorescent z-stack images were acquired using the Opera Phenix High-Content Screening System (PerkinElmer, Waltham, MA).

### Western blotting

MSCs embedded in hydrogels were lysed using radioimmunoprecipitation assay buffer (Fujifilm Wako) supplemented with Halt Protease Inhibitor Cocktail (Thermo Scientific, Waltham, MA) and PhosSTOP Phosphatase Inhibitor (Merck Life Science, Darmstadt, Germany). Total protein concentrations were determined using the Micro BCA Protein Assay Kit (Thermo Scientific, Rockford, IL). Protein samples were then separated by sodium dodecyl sulfate–polyacrylamide Gel electrophoresis using 12% polyacrylamide gels (Bio-Rad, Hercules, CA). Following electrophoresis, proteins were transferred onto iBlot 2 polyvinylidene difluoride membranes (Invitrogen, Carlsbad, CA) using the iBlot 2 Gel Transfer Device (Invitrogen). Membranes were blocked with Blocking One solution (Nacalai Tesque) for 30 min at room temperature to prevent nonspecific binding. Subsequently, membranes were incubated overnight at 4°C with primary antibodies against integrin β1 (#4706, Cell Signaling Technology, Danvers, MA), Rho-associated coiled-coil containing protein kinase 1 (ROCK1; C8F7, #4035, Cell Signaling Technology), and β-actin (D6A8, #8457, Cell Signaling Technology). After washing, membranes were incubated for 1 h at room temperature with horseradish peroxidase–conjugated goat anti-rabbit immunoglobulin G (IgG) secondary antibody (#7074, Cell Signaling Technology). Protein bands were visualized using Western Blot Chemiluminescence Reagent (ImmunoStar, Fujifilm Wako, Richmond, VA) and detected with an imaging system (ChemiDoc XRS^+^, Bio-Rad). To quantify the expression levels of integrin β1 and ROCK1 proteins, band intensities were measured using ImageJ software and normalized to the intensity of the β-actin band.

### Localization of vinculin and F-actin cytoskeleton

Hydrogels were fixed with 4% paraformaldehyde for 24 h and permeabilized with 0.5% Triton X-100 in Tris-buffered saline for 10 min. Following permeabilization, samples were blocked with 10% goat serum at room temperature for 2 h. The hydrogels were then incubated overnight at 4°C with 2 ng/mL monoclonal anti-vinculin antibody diluted in 1% bovine serum albumin. Subsequently, samples were incubated for 1 h at room temperature in the dark with fluorescein isothiocyanate-conjugated goat anti-mouse IgG antibody (EMD Millipore, Burlington, MA) and tetramethylrhodamine-conjugated phalloidin (FAK100, Millipore). Nuclei were counterstained with 4′,6-diamidino-2-phenylindole (DAPI) (FAK100, Millipore) for 10 min at room temperature, followed by thorough washing with PBS containing 0.05% Tween-20. Hydrogels were stored in PBS at 4°C until imaging with a confocal microscope (Leica Stellaris 5, Leica Microsystems GmbH, Wetzlar, Germany). Z-stack images were presented as maximum intensity projections.

### Gene expression analysis

After 24 h and 5 d of culture in the inflammatory medium, hydrogels were collected, snap-frozen in liquid nitrogen, and stored at − 80°C. RNAiso Plus (Takara Bio, Kusatsu, Japan) was added to each hydrogel, and the samples were homogenized using a tissue lyser (Qiagen, Hilden, Germany). Chloroform was then added to facilitate phase separation. Total RNA was extracted using the SV Total RNA Isolation Kit (Promega, Madison, WI), and reverse transcription was carried out using the Transcriptor First Strand cDNA Synthesis Kit (Roche, Indianapolis, IN). Quantitative real-time polymerase chain reaction (qPCR) was performed using the KAPA SYBR Fast qPCR Master Mix (KAPA Biosystem, Cape Town, South Africa) and the StepOnePlus Real-Time PCR System (Applied Biosystems, Waltham, MA). Gene expression was analyzed for COX2, VEGF, IDO1, and TSG6, with GAPDH used as the housekeeping gene. Primer sequences are listed in Table 1.

**Table 1.** Primers used for qPCR (5′ → 3′)

Gene	Forward primer	Reverse primer
GAPDH	CGACAGTCAGCCGCATCTTC	CGCCCAATACGACCAAATCCG
COX2	CGGTGAAACTCTGGCTAGACAG	GCAAACCGTAGATGCTCAGGGA
WEGF	TTGCCTTGCTGCTCTACCTCCA	GATGGCAGTAGCTGCGCTGATA
TSG6	GCGGTGTGTGAATTTGAAGGC	CATCCATCCAGCAGCACAGAC
IDO1	GCCTGATCTCATAGAGTCTGGC	TGCATCCCAGAACTAGACGTGC

### Statistical analysis

Data are presented as mean values, with error bars representing standard deviation of the average value. Statistical analyses were performed using GraphPad Prism9 software (GraphPad Software, Boston, MA) and the significance level was analyzed by one-way ANOVA with a mean comparison using the Tukey method. *p* < 0.05 and *p* < 0.01 were considered statistically significant.

## Results

### MSCs in 3D environments

**Figure 1.**
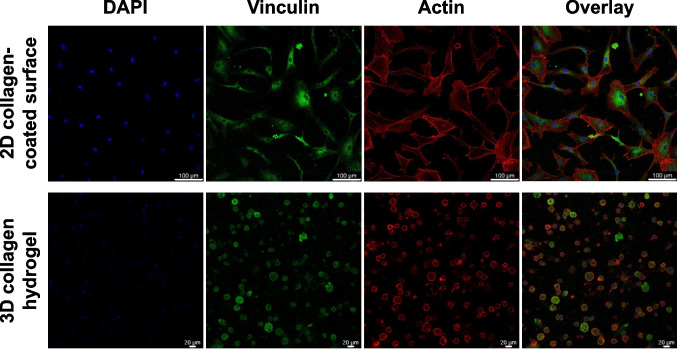
Confocal images of immunofluorescently stained MSCs cultured under inflammatory conditions on 2D collagen-coated surfaces and within 3D collagen hydrogels prepared using 3.5 mg/mL collagen solution. MSCs were seeded at 20,000 cells/cm^2^ for 2D and 3 × 10^6^ cells/mL for 3D cultures. Cells were stained using antibodies to vinculin (*green*) and F-actin (*red*). Nuclei were counterstained with DAPI (*blue*). Z-stack images are presented. *Scale bar*: (*upper row*) 100 μm and (*lower row*) 20 μm.

Confocal microscopy (Fig. 1) revealed that mesenchymal stem cells (MSCs) cultured on a two-dimensional (2D) collagen hydrogel exhibited a flat, large stellate morphology with prominent lamellipodia and filopodia. The cells formed well-defined F-actin stress fibers, and vinculin staining was primarily localized in the cytoplasm. In contrast, MSCs cultured within a 3D collagen hydrogel displayed a more spherical morphology. Focal adhesions showed a distinct distribution pattern, with vinculin localized both at the cell membrane and throughout the cytoplasm. Actin fibers in 3D-cultured cells appeared less mature compared to those in 2D culture.

**Figure 2.**
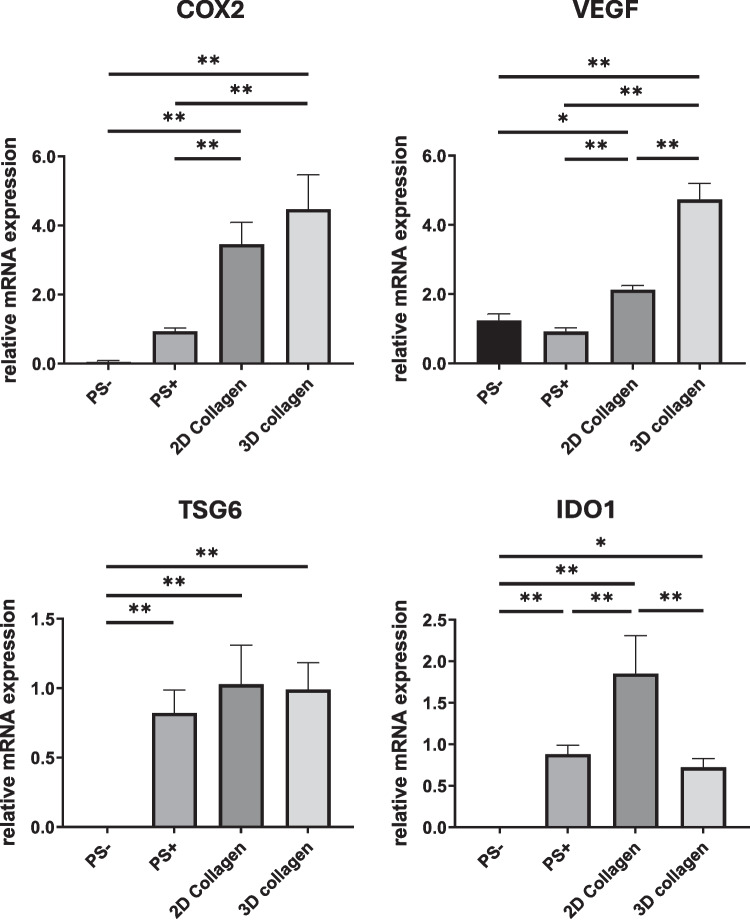
Relative expression levels of COX2, VEGF, TSG6, and IDO1 in MSCs cultured on 2D collagen-coated surfaces and within 3D collagen hydrogels. As controls, MSCs were cultured on polystyrene plate without inflammation stimulation (*PS −*) and under inflammation stimulation (*PS* +). Data are presented as mean ± standard deviation (*n* = 3; *p < 0.05; **p < 0.01).

In order to observe the immunomodulatory response of MSCs incorporated in collagen hydrogels, we used TNF-α and IFN-γ as proinflammatory cytokines to stimulate the inflammatory environment. Both cytokines are known to be mostly involved in inflammation-related diseases and have synergistic interaction (Barcia *et al*. 2011; Ohta *et al*. 2017; Karki *et al*. 2021; Woznicki *et al*. 2021). The immunomodulatory response of MSCs exposed to an inflammatory environment induced by TNF-α and IFN-γ was assessed through quantitative gene expression analysis of trophic and immunomodulatory factors. As shown in Fig. 2, under these inflammatory conditions, MSCs cultured in the 3D collagen hydrogel exhibited significantly elevated level of VEGF Gene expression than those in 2D. The level of COX2 Gene expression was also greater in 3D than 2D, although differences were not significant. In contrast, IDO1 expression was lower in 3D culture than in 2D. Although only one initial seeding density and a single time point were employed, these results suggest that MSCs exhibit distinct immunomodulatory profiles depending on their morphology and the degree of focal adhesion maturation, which are influenced by the dimensionality of the matrix (2D vs. 3D).

### Effect of initial cell seeding density

**Figure 3.**
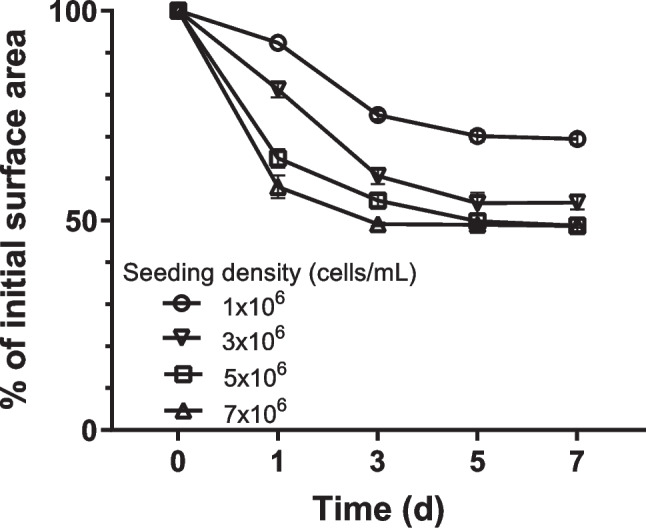
Effect of initial cell seeding density on the contraction of MSC-laden collagen hydrogels prepared with 3.5 mg/mL collagen solution. MSCs were seeded at 1 × 10^6^, 3 × 10^6^, 5 × 10^6^, and 7 × 10.^6^ cells/mL and cultured for 7 d with 10 ng/mL TNF-α and 25 ng/mL IFN-γ. Data are expressed as mean ± standard deviation (*n* = 4 − 5).

As shown in Fig. S1 and Fig. 3, collagen hydrogels contracted over the culture period, and the extent of contraction increased with higher initial cell seeding density. According to previous studies (Ahearne *et al*. 2010; Chieh *et al*. 2010; Hinz *et al*. 2019), the contraction of cell–collagen hydrogel composites is attributed to the contractile forces generated by extracellular matrix components secreted by the cells.

**Figure 4.**
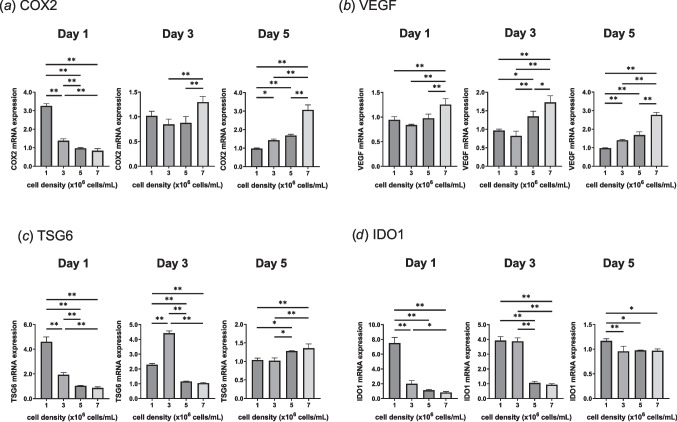
Effect of initial cell seeding density on the expression of immunomodulatory genes: (*a*) COX2, (*b*) VEGF, (*c*) TSG6, and (*d*) IDO1. Hydrogels were prepared using 3.5 mg/mL collagen. MSCs were seeded at 1 × 10^6^, 3 × 10^6^, 5 × 10^6^, and 7 × 10.^6^ cells/mL and cultured for 5 d under inflammatory conditions (10 ng/mL TNF-α and 25 ng/mL IFN-γ). Data are shown as mean ± standard deviation (*n* = 3 − 4; *p < 0.05; **p < 0.01).

Figure 4 presents the expression levels of immunomodulatory genes under TNF-α and IFN-γ stimulation as a function of initial cell seeding density. On day 1, COX2, TSG6, and IDO1 Gene expression were upregulated in hydrogels with lower cell density, whereas an increase in cell density resulted in upregulated VEGF expression. By day 3, COX2 expression shifted to higher levels in the high cell density group, while TSG6 and IDO1 maintained similar expression patterns as observed on day 1. By day 5, the expression levels of both COX2 and VEGF were markedly elevated under high cell density conditions, while TSG6 expression also tended to increase at higher cell density, whereas IDO1 showed less differences across all cell densities. These results demonstrate the dynamic nature of immunomodulatory gene expression in response to varying cell densities. Specifically, a reduced number of cells corresponded to stronger early immunomodulatory responses, while hydrogel contraction became more pronounced with increasing cell density.

### Effect of collagen concentration

**Figure 5.**
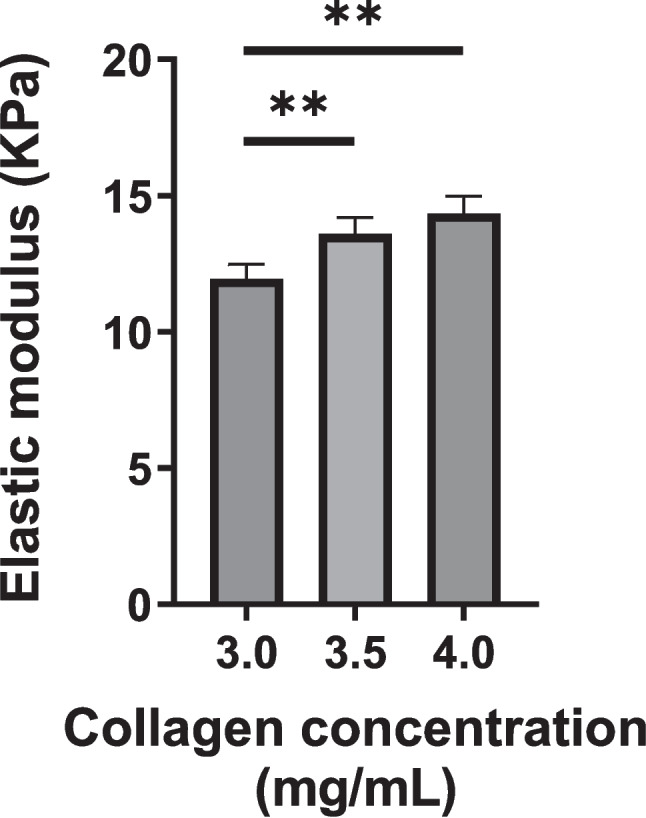
Elastic modulus determined for acellular collagen hydrogels. Data are presented as mean ± standard deviation (*n* = 4 − 5; *p < 0.05; **p < 0.01).

Acellular collagen hydrogels prepared using 3.0, 3.5, and 4.0 mg/mL collagen solutions exhibited comparable stress–strain relationships (Fig. S2). As shown in Fig. 5, the elastic modulus increased with increasing collagen concentration. Collagen hydrogels prepared from a collagen solution incubated at 4°C for 5 min and 3 h prior to gelation showed similar elastic moduli (approximately 13 kPa; Fig. S3). This suggested that the initial stiffness was not significantly influenced by pre-incubation. Therefore, the shorter incubation time was used in subsequent experiments.

**Figure 6.**
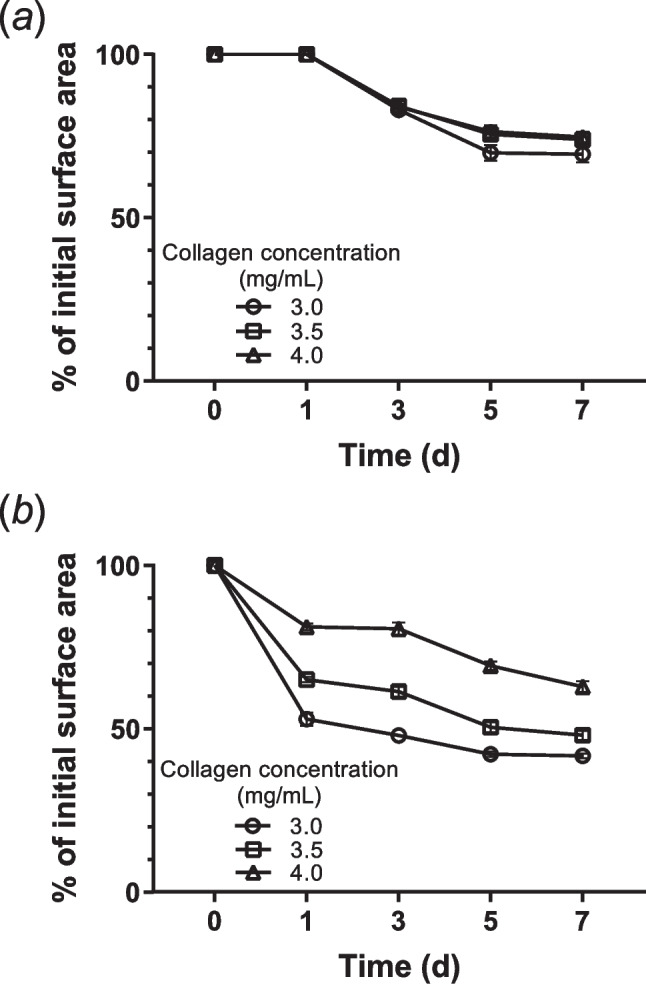
Contraction behavior of MSC-laden collagen hydrogels prepared using 3.0, 3.5, and 4.0 mg/mL collagen solutions. MSCs were seeded at (*a*) 1 × 10^6^ cells/mL and (*b*) 5 × 10.^6^ cells/mL and cultured for 7 d in the presence of 10 ng/mL TNF-α and 25 ng/mL IFN-γ. Data are shown as mean ± standard deviation (*n* = 4 − 5).

MSCs were seeded at densities of 1 × 10^6^ cells/mL (low seeding density) and 5 × 10^6^ cells/mL (high seeding density) into collagen hydrogels composed of 3.0, 3.5, and 4.0 mg/mL collagen. At low seeding density (Fig. 6*a*), negligible contraction was observed on day 1. By day 3, the hydrogels began to contract to a similar extent regardless of collagen concentration, and this contraction continued through day 7. At high seeding density (Fig. 6*b*), hydrogels exhibited markedly greater contraction compared to those with low seeding density. The rate of contraction was inversely related to collagen concentration; softer hydrogels (i.e., 3.0 mg/mL) contracted more rapidly. Within 1 d, the top surface area of hydrogels made with 3.0 mg/mL collagen decreased to 52% of the initial area—clearly more and faster than those made with 3.5 or 4.0 mg/mL collagen.

**Figure 7.**
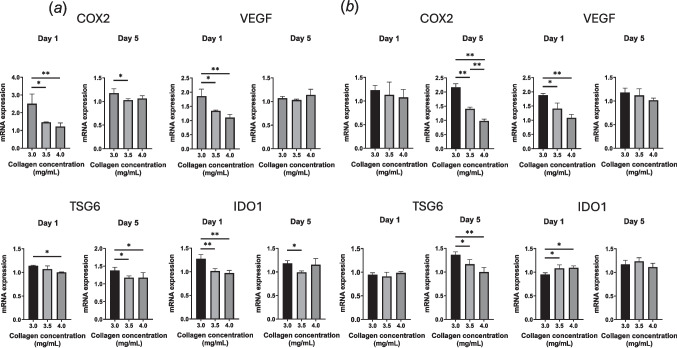
Effect of collagen concentration and cell seeding density on the expression of immunomodulatory genes including COX2, VEGF, TSG6, and IDO1. MSCs were seeded at (*a*) low (1 × 10^6^ cells/mL) and (*b*) high (5 × 10.^6^ cells/mL) densities into hydrogels made with 3.0, 3.5, and 4.0 mg/mL collagen and cultured for 5 d with TNF-α (10 ng/mL) and IFN-γ (25 ng/mL). Data are expressed as mean ± standard deviation (*n* = 3 − 4; *p < 0.05; **p < 0.01).

The expression of immunomodulatory Genes was analyzed on days 1 and 5. At low seeding density (Fig. 7*a*), all immunomodulatory Genes were significantly upregulated in hydrogels with 3.0 mg/mL collagen on day 1, and this trend mostly persisted through day 5. Under high seeding density conditions, hydrogels with 3.5 and 4.0 mg/mL collagen showed increased IDO1 expression on day 1 (Fig. 7*b*), while VEGF expression was higher in the 3.0 mg/mL condition. No significant differences were observed in COX2 and TSG6 expression on day 1. By day 5, COX2, VEGF, TSG6, and IDO1 expression was more pronounced in hydrogels with reduced collagen content. These results suggest that the hydrogel contraction may be associated with the immunomodulatory properties of MSCs.

**Figure 8.**
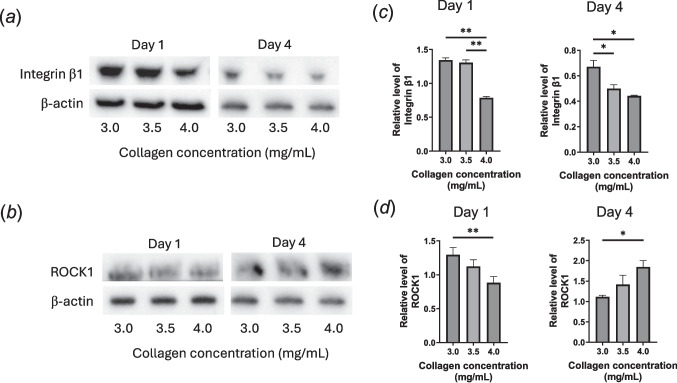
Western blot analysis of (*a*) integrin β_1_ and (*b*) ROCK1 expression in MSCs cultured in collagen hydrogels prepared using 3.0, 3.5, and 4.0 mg/mL collagen. MSCs were seeded at 5 × 10.^6^ cells/mL and cultured for 1 d and 4 d under inflammatory stimulation (10 ng/mL TNF-α and 25 ng/mL IFN-γ). (*c*, *d*) Quantification of expressed (*c*) integrin β_1_ and (*d*) ROCK1. Data are shown as mean ± standard deviation (*n* = 3; *p < 0.05; **p < 0.01).

Western blot analysis (Fig. 8) showed increased expression of integrin β1 in hydrogels with reduced collagen concentrations (Fig. 8*a* and *c*), suggesting enhanced clustering of integrin complexes. ROCK1 kinase expression (Fig. 8*b* and *d*) was elevated in low-concentration hydrogels on day 1, but by day 4, greater expression was observed in high-concentration hydrogels, highlighting the dynamic regulation of ROCK1 activity over time.

**Figure 9.**
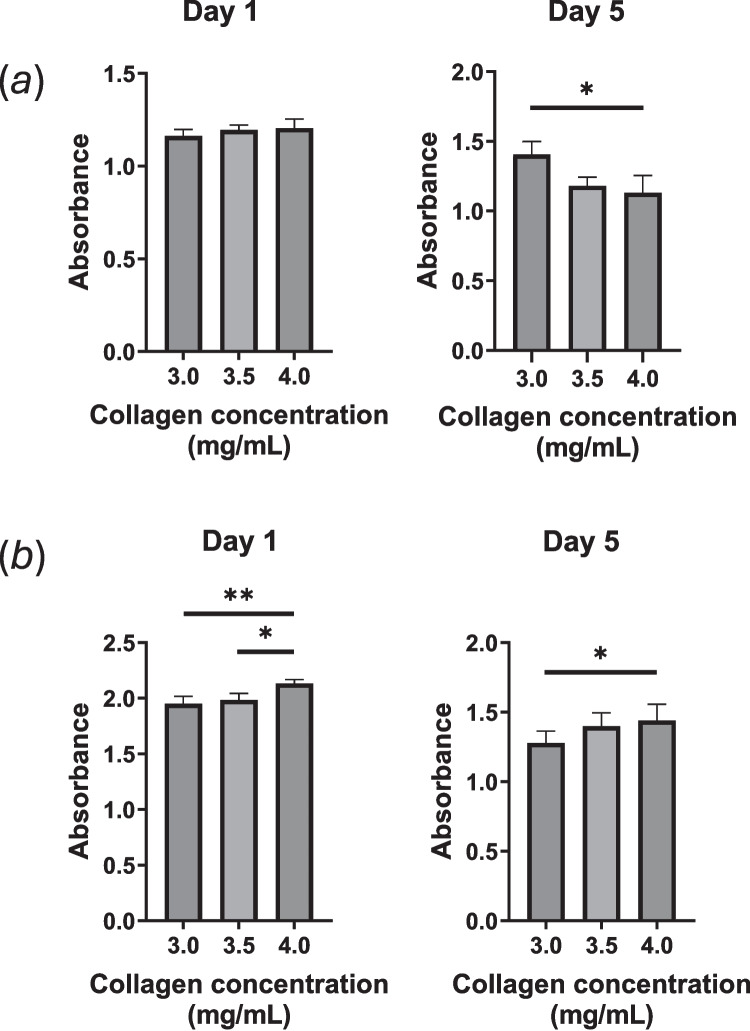
Viability of MSCs embedded in collagen hydrogels prepared using 3.0, 3.5, and 4.0 mg/mL collagen solutions. MSCs were seeded at (*a*) 1 × 10^6^ and (*b*) 5 × 10.^6^ cells/mL and cultured for 5 d in the presence of TNF-α (10 ng/mL) and IFN-γ (25 ng/mL). Data are shown as mean ± standard deviation (*n* = 3 − 4; *p < 0.05; **p < 0.01).

Cell viability in 3D collagen hydrogels of different concentrations is shown in Fig. 9. At low seeding density (Fig. 9*a*), similar cell viability was observed across all conditions on day 1 but higher viability was observed in lower-concentration hydrogels on day 5. Under dense seeding conditions (Fig. 9*b*), cells retained their viability in hydrogels containing more concentrated collagen on both days 1 and 5, with less hydrogel contraction.

## Discussion

**Figure 10.**
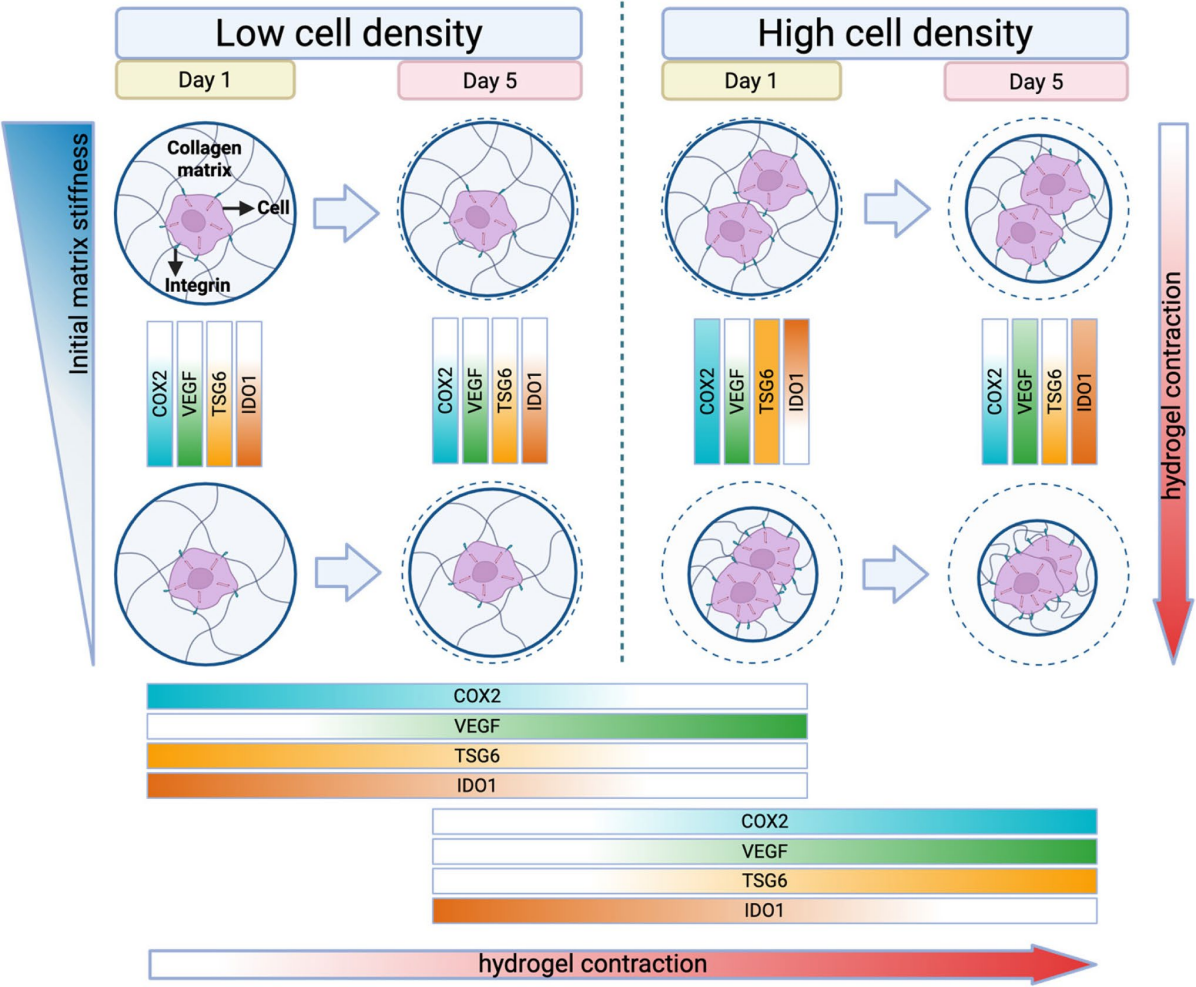
A conceptional illustration depicting the overall findings of this study.

This study investigated how dimensionality, cell seeding density, and collagen concentration influence the immunomodulatory behavior of MSCs embedded in 3D collagen matrices. Our findings demonstrate that these parameters profoundly affect MSC responses by altering the mechanical and structural features of their microenvironment. Figure 10 shows an overview of the key findings from this study.

Compared to traditional 2D cultures, MSCs in 3D collagen matrices exhibited enhanced immunomodulatory activity. This enhancement appears to be driven by the distinct physical context of 3D environments, where MSCs adopt different morphologies and form altered focal adhesions. These changes modulate intracellular signaling pathways, likely due to collagen fiber architecture and mechanical properties between 2D and 3D systems.

We found that both cell density and collagen concentration modulate the degree of hydrogel contraction, which in turn influences immunomodulatory gene expression.

At low seeding densities, MSCs induced modest matrix contraction, correlating with increased expression of immunoregulatory genes. In contrast, high seeding densities in softer, low-collagen hydrogels led to rapid and pronounced contraction, which may have increased local matrix stiffness due to fiber compaction. Higher cell numbers increased the attachment points to the collagen matrix, and the amount of force that could be generated to contract the hydrogels also increased. Lower initial collagen concentration resulted in less mechanical resistance during cellular contraction of the matrix, hence facilitating a faster and easier contraction. This observation aligns with previous reports (Ahearne *et al*. 2010) describing strain-induced stiffening in contracting collagen matrices.

Mechanical characterization of the hydrogels confirmed that higher collagen concentrations result in increased stiffness. Notably, low-density cultures in stiff matrices showed little contraction initially, allowing us to isolate the effect of stiffness without the confounding influence of cell-induced remodeling. Under these conditions, softer hydrogels supported greater immunomodulatory Gene expression, consistent with prior 2D studies showing that compliant substrates enhance immunomodulatory potential (Ji *et al*. 2019; Yoshii *et al*. 2024).

MSCs, being anchorage-dependent, sense mechanical cues through integrin-mediated focal adhesions, which link the extracellular matrix to the actin cytoskeleton. These structures transduce mechanical signals via Ras homolog family member A (RhoA)/ROCK pathways, regulating actin dynamics, gene expression, and cell fate (Krouwels *et al*. 2018; Guan *et al*. 2023). ROCK1, a downstream effector of the small GTPase RhoA, acts as a key regulator of the actin cytoskeleton by phosphorylating myosin regulatory light chain (Shi *et al*. 2013) and altering myosin II activity that generates cell traction force (Peng *et al*. 2019).

ROCK1 mechanosignaling through the actin cytoskeleton is also responsible for activation of YAP/TAZ nuclear localization (Ou *et al*. 2021). In the nucleus, YAP/TAZ interacts with transcription factors, most notably the transcriptional enhanced associate domain (TEAD) family, to bind to specific DNA sequences and regulate gene expression (Pocaterra *et al*. 2020; Esposito *et al*. 2022). A study by Yoshii *et al*. (2024) showed that the mechanosignaling YAP/TAZ-TEAD axis negatively regulates the immunomodulatory properties in MSCs. These may elucidate that the elevated ROCK1 levels in stiffer collagen hydrogels lead to the downregulation of the immunomodulatory genes expression.

The dynamic nature of the collagen matrix further complicates the relationship between mechanics and cell behavior. Collagen gels exhibit strain-stiffening and viscoelastic stress relaxation, especially at low concentrations. As shown by Nam *et al*. (2016), collagen matrices stiffen under rapid deformation but behave as viscous materials over time. At greater deformations, the hydrogel becomes stiffer but then flows more rapidly to relax this increase in stiffness. These mechanical transitions likely affect mechanotransduction in MSCs by altering how the cells detect and adapt to matrix stiffness during contraction and stress relaxation. Our results suggest that changes in matrix stiffness and viscoelasticity, rather than simply the number of adhesion points, predominantly regulate MSC responses to inflammatory stimuli, as evidenced by variable gene expression profiles across different matrix conditions. Moreover, the expression of COX2 and VEGF is more sensitive to the dynamic alterations in the mechanical characteristics of the extracellular matrix compared to TSG6 and IDO1.

Importantly, hydrogel contraction also influenced cell viability. At low seeding densities, where contraction was minimal, viability remained high regardless of collagen concentration. However, under high seeding densities in soft matrices—conditions that produced strong contraction—cell viability decreased. This may result from increased mechanical stress and localized crowding, which could impair nutrient diffusion and increase cellular strain. These findings suggest a trade-off between enhancing immunomodulatory activity and maintaining cell viability under conditions of high contraction.

Finally, MSCs stimulated with TNF-α and IFN-γ—proinflammatory cytokines known to act synergistically—responded not only to biochemical signals but also to physical cues from the matrix. The combined influence of cytokine exposure and matrix mechanics dynamically shaped MSC immunomodulatory behavior.

## Conclusion

This study demonstrates that the immunomodulatory behavior of MSCs is strongly influenced by their 3D microenvironment. Factors such as matrix stiffness, collagen concentration, and cell density affect MSC morphology, mechanotransduction, and gene expression. Softer, less-contracted matrices enhance immunomodulatory activity, while excessive contraction under high cell density can reduce cell viability. These findings emphasize the need to balance mechanical cues to optimize MSC function and survival.

## Supplementary Information

Below is the link to the electronic supplementary material.

ESM 1 (PDF 477 KB)

## Data Availability

All data generated or analyzed during this study are included in this published article.

## References

[CR1] Ahearne M, Wilson SL, Liu KK, Rauz S, El Haj AJ, Yang Y (2010) Influence of cell and collagen concentration on the cell-matrix mechanical relationship in a corneal stroma wound healing model. Exp Eye Res 91:584–591. 10.1016/j.exer.2010.07.013 20678499 10.1016/j.exer.2010.07.013

[CR2] Ankrum JA, Ong JF, Karp JM (2014) Mesenchymal stem cells: immune evasive, not immune privileged. Nat Biotechnol 32:252–260. 10.1038/nbt.2816 24561556 10.1038/nbt.2816 PMC4320647

[CR3] Barcia C, Ros CM, Annese V, Gómez A, Ros-Bernal F, Aguado-Year C, Martínez-Paǵn ME, De Pablos V, Fernandez-Villalba E, Herrero MT (2011) IFN-γ signaling, with the synergistic contribution of TNF-α, mediates cell specific microglial and astroglial activation in experimental models of Parkinson’s disease. Cell Death Dis 2:e142. 10.1038/cddis.2011.17 21472005 10.1038/cddis.2011.17 PMC3122054

[CR4] Bernardo ME, Fibbe WE (2013) Mesenchymal stromal cells: sensors and switchers of inflammation. Cell Stem Cell 13:392–402. 10.1016/j.stem.2013.09.006 24094322 10.1016/j.stem.2013.09.006

[CR5] Burand AJ, Di L, Boland LK, Boyt DT, Schrodt MV, Santillan DA, Ankrum JA (2020) Aggregation of human mesenchymal stromal cells eliminates their ability to suppress human T cells. Front Immunol 11:143. 10.3389/fimmu.2020.00143 32158443 10.3389/fimmu.2020.00143 PMC7052295

[CR6] Burdick JA, Mauck RL, Gerecht S (2016) To serve and protect: hydrogels to improve stem cell-based therapies. Cell Stem Cell 18:13–15. 10.1016/j.stem.2015.12.004 26748751 10.1016/j.stem.2015.12.004

[CR7] Chieh HF, Sun Y, Liao JD, Su FC, Zhao C, Amadio PC, An KN (2010) Effects of cell concentration and collagen concentration on contraction kinetics and mechanical properties in a bone marrow stromal cell-collagen construct. J Biomed Mater Res A 93:1132–1139. 10.1002/jbm.a.32606 19768794 10.1002/jbm.a.32606 PMC3900770

[CR8] Chung H, Oh S, Shin HW, Lee Y, Lee H, Seok SH (2021) Matrix stiffening enhances DNCB-induced IL-6 secretion in keratinocytes through activation of ERK and PI3K/Akt pathway. Front Immunol 12:759992. 10.3389/fimmu.2021.759992 34858412 10.3389/fimmu.2021.759992 PMC8631934

[CR9] Esposito D, Pant I, Shen Y, Qiao RF, Yang X, Bai Y, Jin J, Poulikakos PI, Aaronson SA (2022) ROCK1 mechano-signaling dependency of human malignancies driven by TEAD/YAP activation. Nat Commun 13:703. 10.1038/s41467-022-28319-3 35121738 10.1038/s41467-022-28319-3 PMC8817028

[CR10] Farini A, Sitzia C, Erratico S, Meregalli M, Torrente Y (2014) Clinical applications of mesenchymal stem cells in chronic diseases. Stem Cells Int 2014:306573. 10.1155/2014/306573 24876848 10.1155/2014/306573 PMC4021690

[CR11] Galland S, Vuille J, Martin P, Letovanec I, Caignard A, Fregni G, Stamenkovic I (2017) Tumor-derived mesenchymal stem cells use distinct mechanisms to block the activity of natural killer cell subsets. Cell Rep 20:2891–2905. 10.1016/j.celrep.2017.08.089 28930684 10.1016/j.celrep.2017.08.089

[CR12] Ge Q, Zhang H, Hou J, Wan L, Cheng W, Wang X, Dong D, Chen C, Xia J, Guo J, Chen X, Wu X (2018) VEGF secreted by mesenchymal stem cells mediates the differentiation of endothelial progenitor cells into endothelial cells via paracrine mechanisms. Mol Med Rep 17:1667–1675. 10.3892/mmr.2017.8059 29138837 10.3892/mmr.2017.8059 PMC5780109

[CR13] Guan G, Cannon RD, Coates DE, Mei L (2023) Effect of the Rho-kinase/ROCK signaling pathway on cytoskeleton components. Genes (Basel) 14:272. 10.3390/genes14020272 36833199 10.3390/genes14020272 PMC9957420

[CR14] Harrell CR, Djonov V, Volarevic V (2021) The cross-talk between mesenchymal stem cells and immune cells in tissue repair and regeneration. Int J Mol Sci 22:2472. 10.3390/ijms22052472 33804369 10.3390/ijms22052472 PMC7957490

[CR15] He X, Wang Q, Zhao Y, Zhang H, Wang B, Pan J, Li J, Yu H, Wang L, Dai J, Wang D (2020) Effect of intramyocardial grafting collagen scaffold with mesenchymal stromal cells in patients with chronic ischemic heart disease: a randomized clinical trial. JAMA Netw Open 3:e2016236. 10.1001/jamanetworkopen.2020.16236 32910197 10.1001/jamanetworkopen.2020.16236 PMC7489863

[CR16] Hinz B, McCulloch CA, Coelho NM (2019) Mechanical regulation of myofibroblast phenoconversion and collagen contraction. Exp Cell Res 379:119–128. 10.1016/j.yexcr.2019.03.027 30910400 10.1016/j.yexcr.2019.03.027

[CR17] Huang Y, Li X, Yang L (2022) Hydrogel encapsulation: taking the therapy of mesenchymal stem cells and their derived secretome to the next level. Front Bioeng Biotechnol 10:1–9. 10.3389/fbioe.2022.859927 10.3389/fbioe.2022.859927 PMC9011103 35433656

[CR18] Ji Y, Li J, Wei Y, Gao W, Fu X, Wang Y (2019) Substrate stiffness affects the immunosuppressive and trophic function of hMSCs: via modulating cytoskeletal polymerization and tension. Biomater Sci 7:5292–5300. 10.1039/c9bm01202h 31612176 10.1039/c9bm01202h

[CR19] Jiang W, Xu J (2020) Immune modulation by mesenchymal stem cells. Cell Prolif 53:e12712. 10.1111/cpr.12712 31730279 10.1111/cpr.12712 PMC6985662

[CR20] Karki R, Sharma BR, Tuladhar S, Williams EP, Zalduondo L, Samir P, Zheng M, Sundaram B, Banoth B, Malireddi RKS, Schreiner P, Neale G, Vogel P, Webby R, Jonsson CB, Kanneganti TD (2021) Synergism of TNF-α and IFN-γ triggers inflammatory cell death, tissue damage, and mortality in SARS-CoV-2 infection and cytokine shock syndromes. Cell 184:149–168. 10.1016/j.cell.2020.11.025 33278357 10.1016/j.cell.2020.11.025 PMC7674074

[CR21] Kozhukharova I, Minkevich N, Alekseenko L, Domnina A, Lyublinskaya O (2022) Paracrine and autocrine effects of VEGF are enhanced in human eMSC spheroids. Int J Mol Sci 23:14324. 10.3390/ijms232214324 36430800 10.3390/ijms232214324 PMC9695450

[CR22] Krouwels A, Melchels FPW, van Rijen MHP, Ten Brink CBM, Dhert WJA, Cumhur Öner F, Tryfonidou MA, Creemers LB (2018) Focal adhesion signaling affects regeneration by human nucleus pulposus cells in collagen- but not carbohydrate-based hydrogels. Acta Biomater 66:238–247. 10.1016/j.actbio.2017.11.029 29174589 10.1016/j.actbio.2017.11.029

[CR23] Levit RD, Landázuri N, Phelps EA, Brown ME, García AJ, Davis ME, Joseph G, Long R, Safley SA, Suever JD, Lyle AN, Weber CJ, Taylor WR (2013) Cellular encapsulation enhances cardiac repair. J Am Heart Assoc 2:e000367. 10.1161/JAHA.113.000367 24113327 10.1161/JAHA.113.000367 PMC3835246

[CR24] Lu D, Xu Y, Liu Q, Zhang Q (2021) Mesenchymal stem cell-macrophage crosstalk and maintenance of inflammatory microenvironment homeostasis. Front Cell Dev Biol 9:681171. 10.3389/fcell.2021.681171 34249933 10.3389/fcell.2021.681171 PMC8267370

[CR25] Marquardt LM, Heilshorn SC (2016) Design of injectable materials to improve stem cell transplantation. Curr Stem Cell Rep 2:207–220. 10.1007/s40778-016-0058-0 28868235 10.1007/s40778-016-0058-0 PMC5576562

[CR26] Nam S, Hu KH, Butte MJ, Chaudhuri O (2016) Strain-enhanced stress relaxation impacts nonlinear elasticity in collagen gels. Proc Natl Acad Sci U S A 113:5492–5497. 10.1073/pnas.1523906113 27140623 10.1073/pnas.1523906113 PMC4878492

[CR27] Ohta K, Naruse T, Kato H, Ishida Y, Nakagawa T, Ono S, Shigeishi H, Takechi M (2017) Differential regulation by IFN-γ on TNF-α-induced chemokine expression in synovial fibroblasts from temporomandibular joint. Mol Med Rep 16:6850–6857. 10.3892/mmr.2017.7432 28901435 10.3892/mmr.2017.7432

[CR28] Ou W, Xu W, Liu F, Guo Y, Huang Z, Feng T, Liu CY, Du P (2021) Increased expression of yes-associated protein/YAP and transcriptional coactivator with PDZ-binding motif/TAZ activates intestinal fibroblasts to promote intestinal obstruction in Crohn’s disease. eBioMedicine 69:103452. 10.1016/j.ebiom.2021.103452 34186485 10.1016/j.ebiom.2021.103452 PMC8243379

[CR29] Peng Y, Chen Z, Chen Y, Li S, Jiang Y, Yang H, Wu C, You F, Zheng C, Zhu J, Tan Y, Qin X, Liu Y (2019) ROCK isoforms differentially modulate cancer cell motility by mechanosensing the substrate stiffness. Acta Biomater 88:86–101. 10.1016/j.actbio.2019.02.015 30771534 10.1016/j.actbio.2019.02.015

[CR30] Pittenger MF, Discher DE, Péault BM, Phinney DG, Hare JM, Caplan AI (2019) Mesenchymal stem cell perspective: cell biology to clinical progress. NPJ Regen Med 4:22. 10.1038/s41536-019-0083-6 31815001 10.1038/s41536-019-0083-6 PMC6889290

[CR31] Pocaterra A, Romani P, Dupont S (2020) YAP/TAZ functions and their regulation at a glance. J Cell Sci 133:jcs230425. 10.1242/jcs.230425 31996398 10.1242/jcs.230425

[CR32] Prockop DJ, Oh JY (2012) Mesenchymal stem/stromal cells (MSCs): role as guardians of inflammation. Mol Ther 20:14–20. 10.1038/mt.2011.211 22008910 10.1038/mt.2011.211 PMC3255583

[CR33] Rodríguez-Fuentes DE, Fernández-Garza LE, Samia-Meza JA, Barrera-Barrera SA, Caplan AI, Barrera-Saldaña HA (2021) Mesenchymal stem cells current clinical applications: a systematic review. Arch Med Res 52:93–101. 10.1016/j.arcmed.2020.08.00 32977984 10.1016/j.arcmed.2020.08.006

[CR34] Sagaradze GD, Basalova NA, Efimenko AY, Tkachuk VA (2020) Mesenchymal stromal cells as critical contributors to tissue regeneration. Front Cell Dev Biol 8:576176. 10.3389/fcell.2020.576176 33102483 10.3389/fcell.2020.576176 PMC7546871

[CR35] Sarrigiannidis SO, Rey JM, Dobre O, González-García C, Dalby MJ, Salmeron-Sanchez M (2021) A tough act to follow: collagen hydrogel modifications to improve mechanical and growth factor loading capabilities. Mater Today Bio 10:100098. 10.1016/j.mtbio.2021.100098 33763641 10.1016/j.mtbio.2021.100098 PMC7973388

[CR36] Shi J, Wu X, Surma M, Vemula S, Zhang L, Yang Y, Kapur R, Wei L (2013) Distinct roles for ROCK1 and ROCK2 in the regulation of cell detachment. Cell Death Dis 4:e483. 10.1038/cddis.2013.10 23392171 10.1038/cddis.2013.10 PMC3734810

[CR37] Troy E, Tilbury MA, Power AM, Wall JG (2021) Nature-based biomaterials and their application in biomedicine. Polymers 13:332. 10.3390/polym13193321 34641137 10.3390/polym13193321 PMC8513057

[CR38] Vasandan AB, Jahnavi S, Shashank C, Prasad P, Kumar A, Prasanna SJ (2016) Human mesenchymal stem cells program macrophage plasticity by altering their metabolic status via a PGE2-dependent mechanism. Sci Rep 6:38308. 10.1038/srep38308 27910911 10.1038/srep38308 PMC5133610

[CR39] Wong JKU, Mehta A, Vũ TT, Yeo GC (2023) Cellular modifications and biomaterial design to improve mesenchymal stem cell transplantation. Biomater Sci 11:4752–4773. 10.1039/D3BM00376K 37233031 10.1039/d3bm00376k

[CR40] Woznicki JA, Saini N, Flood P, Rajaram S, Lee CM, Stamou P, Skowyra A, Bustamante-Garrido M, Regazzoni K, Crawford N, McDade SS, Longley DB, Aza-Blanc P, Shanahan F, Zulquernain SA, McCarthy J, Melgar S, McRae BL, Nally K (2021) TNF-α synergises with IFN-γ to induce caspase-8-JAK1/2-STAT1-dependent death of intestinal epithelial cells. Cell Death Dis 12:864. 10.1038/s41419-021-04151-3 34556638 10.1038/s41419-021-04151-3 PMC8459343

[CR41] Wu X, Jiang J, Gu Z, Zhang J, Chen Y, Liu X (2020) Mesenchymal stromal cell therapies: immunomodulatory properties and clinical progress. Stem Cell Res Ther 11:345. 10.1186/s13287-020-01855-9 32771052 10.1186/s13287-020-01855-9 PMC7414268

[CR42] Yoshii H, Kajiya M, Yoshino M, Morimoto S, Horikoshi S, Tari M, Motoike S, Iwata T, Ouhara K, Ando T, Yoshimoto T, Shintani T, Mizuno N (2024) Mechanosignaling YAP/TAZ-TEAD axis regulates the immunomodulatory properties of mesenchymal stem cells. Stem Cell Rev Rep 20:347–361. 10.1007/s12015-023-10646-7 37917410 10.1007/s12015-023-10646-7

[CR43] Zangi L, Margalit R, Reich-Zeliger S, Bachar-Lustig E, Beilhack A, Negrin R, Reisner Y (2009) Direct imaging of immune rejection and memory induction by allogeneic mesenchymal stromal cells. Stem Cells 27:2865–2874. 10.1002/stem.217 19750539 10.1002/stem.217

[CR44] Zhang Q, Nguyen P, Burrell JC, Zeng J, Shi S, Shanti RM, Kulischak G, Cullen DK, Le AD (2021) Harnessing 3D collagen hydrogel-directed conversion of human GMSCs into SCP-like cells to generate functionalized nerve conduits. NPJ Regen Med 6:59. 10.1038/s41536-021-00170-y PMC8484485 34593823

