# Supplementary material for: Immunomodulatory properties of mesenchymal stem cells within three-dimensional collagen matrices
Source: In Vitro Cell Dev Biol Anim. 2025 Sep 15;61(9):1158–70. doi: 10.1007/s11626-025-01109-z (PMC12628475; doi:10.1007/s11626-025-01109-z)
Supplement: Supplementary file 1 — (PDF 477 KB) [file 11626_2025_1109_MOESM1_ESM.pdf]

Supplementary Data

Immunomodulatory Properties of Mesenchymal Stem Cells within Three-Dimensional Collagen Matrices

Yenny Yustisia[1,2] · Koichi Kato[1]*

[1] Department of Biomaterials, Graduate School of Biomedical and Health Sciences, Hiroshima University, 1-2-3, Kasumi, Minami-ku, Hiroshima 734-8553, Japan

[2] Department of Oral Biology, Faculty of Dentistry, University of Jember, Kalimantan 37 Jember, Indonesia

*Corresponding author

E-mail: kokato@hiroshima-u.ac.jp

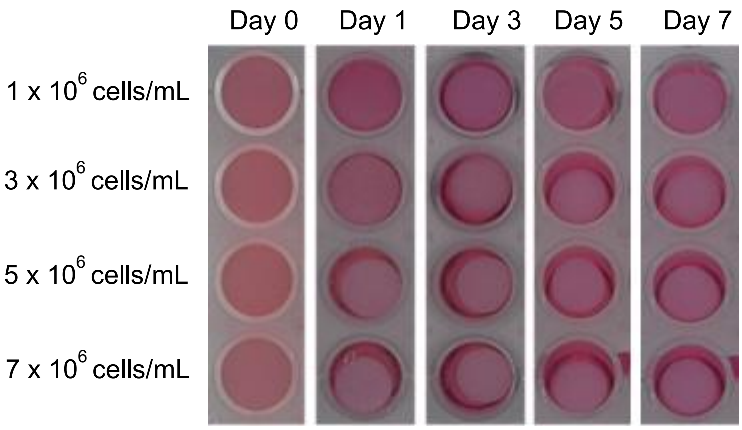

Figure S1. Effect of initial cell seeding density on the contraction of MSC-laden collagen hydrogels. Representative images of collagen hydrogel constructs are shown. Hydrogels were prepared using 3.5 mg/mL collagen solution. MSCs were seeded at 1×10^6, 3×10^6, 5×10^6, and 7×10^6 cells/mL and cultured for 7 d with 10 ng/mL TNF-α and 25 ng/mL IFN-γ.

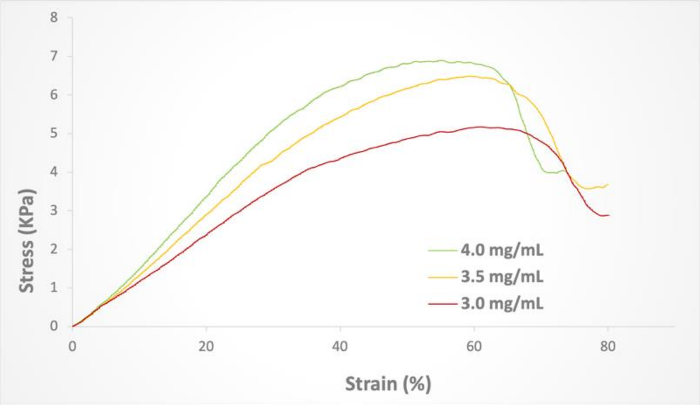

Figure S2. Representative stress–strain curves for hydrogels prepared with 3.0, 3.5, and 4.0 mg/mL collagen concentrations.

(*a*) (*b*)

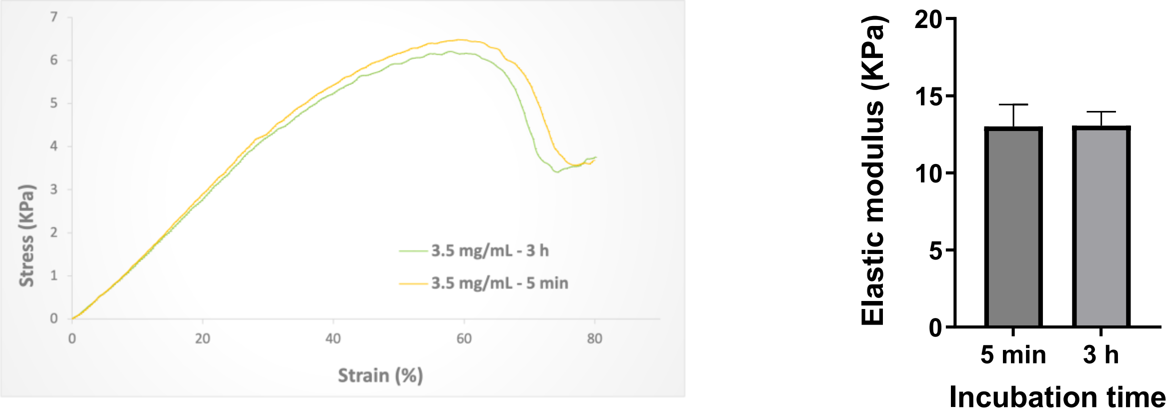

Figure S3. Mechanical testing of acellular collagen hydrogels prepared by incubating 3.5 mg/mL collagen solution for 5 min or 3 h at 4 °C prior to gelation. (*a*) Representative stress–strain curves. (b) Elastic modulus determined from the from the stress–strain curves. Data are presented as mean ± standard deviation (n = 5)

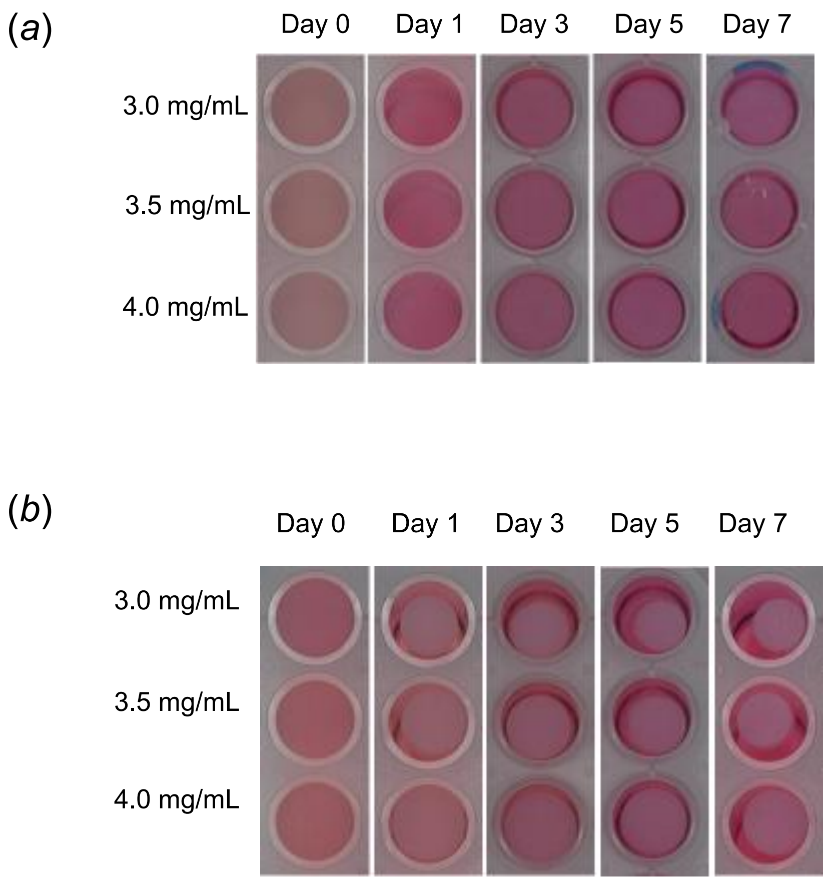

Figure S4. Optical images of MSC-laden collagen hydrogels prepared using 3.0, 3.5, and 4.0 mg/mL collagen solutions. MSCs were seeded at (*a*) 1×10^6 cells/mL and (b) 5×10^6 cells/mL and cultured for 7 d in the presence of 10 ng/mL TNF-α and 25 ng/mL IFN-γ.

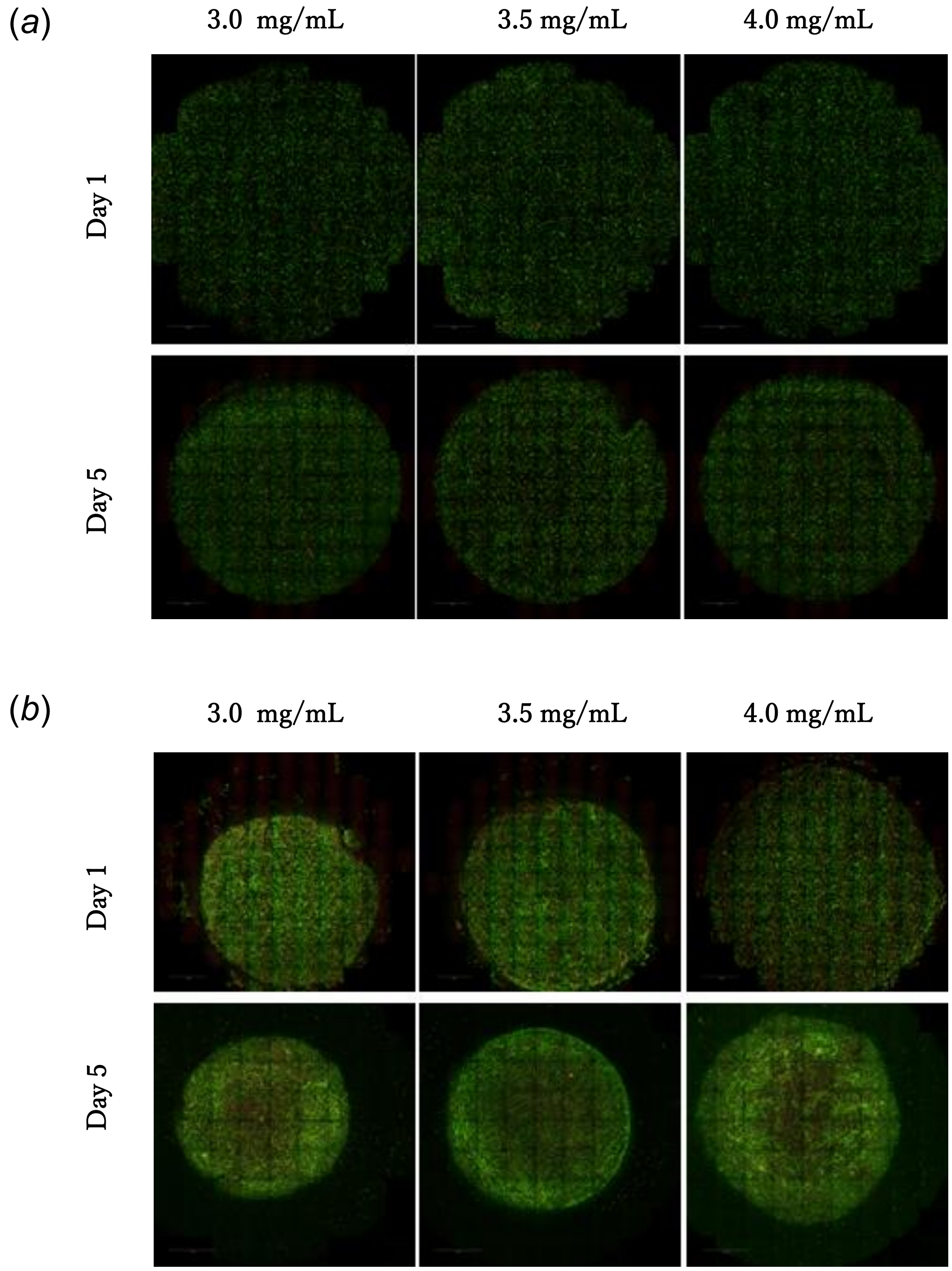

Figure S5. Representative images of MSC viability in collagen hydrogels assessed using Calcein-AM/propidium iodide (PI) staining. Collagen hydrogels were prepared with concentrations of 3.0, 3.5, and 4.0 mg/mL and seeded with MSCs at densities of (a) 1×10^6 cells/mL and (b) 5×10^6 cells/mL. Constructs were cultured for 5 days in the presence of 10 ng/mL TNF-α and 25 ng/mL IFN-γ.
